# Inhibitory effect and underlying mechanism of cinnamon and clove essential oils on *Botryosphaeria dothidea* and *Colletotrichum gloeosporioides* causing rots in postharvest bagging-free apple fruits

**DOI:** 10.3389/fmicb.2023.1109028

**Published:** 2023-02-27

**Authors:** Dan Wang, Guiping Wang, Jinzheng Wang, Hao Zhai, Xiaomin Xue

**Affiliations:** Shandong Institute of Pomology, Shandong Academy of Agricultural Sciences, Tai'an, China

**Keywords:** bagging-free apple, post-harvest pathogens, essential oil, cell membrane, transcriptome

## Abstract

Bagging-free apple is more vulnerable to postharvest disease, which severely limits the cultivation pattern transformation of the apple industry in China. This study aimed to ascertain the dominant pathogens in postharvest bagging-free apples, to evaluate the efficacy of essential oil (EO) on inhibition of fungal growth, and to further clarify the molecular mechanism of this action. By morphological characteristics and rDNA sequence analyses, *Botryosphaeria dothidea* (*B. dothidea*) and *Colletotrichum gloeosporioides* (*C. gloeosporioides*) were identified as the main pathogens isolated from decayed bagging-free apples. Cinnamon and clove EO exhibited high inhibitory activities against mycelial growth both in vapor and contact phases under *in vitro* conditions. EO vapor at a concentration of 60 μL L^−1^ significantly reduced the incidence and lesion diameter of inoculated decay *in vivo*. Observations using a scanning electron microscope (SEM) and transmission electron microscope (TEM) revealed that EO changed the mycelial morphology and cellular ultrastructure and destroyed the integrity and structure of cell membranes and major organelles. Using RNA sequencing and bioinformatics, it was demonstrated that clove EO treatment impaired the cell membrane integrity and biological function *via* downregulating the genes involved in the membrane component and transmembrane transport. Simultaneously, a stronger binding affinity of *trans*-cinnamaldehyde and eugenol with CYP51 was assessed by *in silico* analysis, attenuating the activity of this ergosterol synthesis enzyme. Moreover, pronounced alternations in the oxidation/reduction reaction and critical materials metabolism of clove EO-treated *C. gloeosporioides* were also observed from transcriptomic data. Altogether, these findings contributed novel antimicrobial cellular and molecular mechanisms of EO, suggesting its potential use as a natural and useful preservative for controlling postharvest spoilage in bagging-free apples.

## Introduction

Apple is one of the most important temperate tree fruits in the world. China is the leader in apple production and accounts for more than 50% of the total yield. Pre-harvest bagging has been conventionally practiced for apple cultivation in China, Japan, and Australia in order to improve fruit appearance and increase market value (Fallahi et al., 2001), but such treatment alters the microenvironment for apple development, leading to multiple effects on internal quality such as reduced phenolic compounds and faded flavor (Arakawa et al., 1994; Chen et al., 2012; Feng et al., 2014). In recent years, there has been growing interests in examining the potential of non-bagging patterns due to the decline of fruit inner quality and the increase in labor force cost and ecological pollution. It has been recognized that the bagging-free cultivation pattern is an inevitable trend in the apple industry development in China.

Nevertheless, under the bagging-free cultivation mode, apples are more vulnerable to insect pests and diseases resulting from the lack of bag protection. Although through monitoring and forecasting, combined with biological management and precise pesticide application, the incidence of pre-harvest pests and diseases is controlled below 1% (Zhai et al., 2019), bagging-free apples show higher susceptibility to postharvest disease damage mainly caused by latent fungal pathogens infection in the field or cold chain handling process, which limits good fruit quality and shortens the shelf life of fresh apple fruit during storage, resulting in significant economic losses. At present, relevant studies on pathological research during the conservation period, especially fungal decay that occurred in bagging-free apples, have seldom been reported.

Chemically synthetic fungicides, like difenoconazole, are commonly used for the postharvest control of apple spoilage and pathogenic fungus. In the last decade, there is strong public and scientific controversy about the application of pesticides because of their hazardous side impacts on the environment and human health such as residual toxicity and fungicide-resistance development (Holmes et al., 2016; Hofer, 2019). In this case, consumer demands for organically produced fruit led to the extensive use of naturally derived preservatives. Essential oils (EOs) are secondary metabolites directly extracted from aromatic and medicinal plants, containing a variety of substances called “phytochemicals” and having a natural or avirulent image. Due to the abundant bioactive chemical components, EOs are endowed with remarkable antimicrobial and antioxidative activities, which enable them to effectively defend against foodborne pathogens and extend the storage life of fresh products (Kwon et al., 2017; Alanazi et al., 2018; Ju et al., 2018; Almeida et al., 2019). In general, two application methods may be used for the inhibition of postharvest mold: (i) fumigation solution in which less EO is used due to its high volatility, and (ii) directly contact solution by agar or broth diffusion (Sivakumar and Bautista-Baños, 2014; Wang et al., 2019).

Essential oils exhibited a great variety of the microbial inhibitory spectrum due to their complex chemical composition and diverse mode of action. Cinnamon and clove EO are promising natural fungicides widely used for the control of foodborne pathogens and spoilage microorganisms, and cinnamaldehyde and eugenol are the major active ingredients of them, respectively (Tu et al., 2018; Dávila-Rodríguez et al., 2019; El amrani et al., 2019). Recently, the antimicrobial effect of cinnamon and clove EO has been widely researched. Duduk et al. (2015) found that cinnamon EO had good inhibitory effects against *Colletotrichum acutatum* isolated from strawberry anthracnose. Castellanos et al. (2020) demonstrated that clove EO could reduce the growth of *Aspergillus nige*r from 50 to 70% and *Fusarium oxysporum* to 40% and provide an alternative solution to the use of hazardous chemical fungicides for the postharvest treatment of tomato during storage and transportation. Khaleque et al. (2016) reported that high concentrations of cinnamon and clove EOs could inhibit *Listeria monocytogenes* in ground beef meat and improve the safety of ground beef products. *Botryosphaeria dothidea* (*B. dothidea*) and *Colletotrichum gloeosporioides* (*C. gloeosporioides*) can infect apple fruit before and after picking, thus, resulting in reduced apple yield (Moreira et al., 2019; Yu et al., 2022). EOs or components have been demonstrated as useful effective antifungal agents against postharvest *B. dothidea* and *C. gloeosporioides*. Carvacrol appeared to evidently inhibit the mitochondrial activity and respiration rate of *B. dothidea* and could be a very useful EO compound for controlling postharvest rot soft in kiwifruit (Li J. et al., 2021). Rabari et al. (2018) tested the inhibitory activities of 75 EOs against *C. gloeosporioides* isolated from the infected mango, and four EOs showed remarkably higher antifungal efficacy.

Although many studies reported EO's antimicrobial action, very few have focused on the mechanism underlying these effects and the investigation at the cellular or molecular level is yet to be explored. The fungal cell membrane was the main target for the fungistatic action of EOs due to their lipophilicity (Burt, 2004). In the previous study, it was suggested that an increase in membrane permeability and subsequent release of cellular material might be responsible for EO's antifungal ability (Shao et al., 2013). Lanosterol 14α-demethylase (also known as CYP51) served as a rate-limiting enzyme that can regulate the rate and quantity of ergosterol produced in the fungal cell membrane (Monk et al., 2020). Azole class of antifungal drugs inhibited CYP51, and subsequently, researchers considered CYP51 as the most attractive protein target to develop antifungal drugs (Sun et al., 2019; Dong et al., 2020). However, there is a piece of scattered information where phytochemicals could inhibit CYP51. Several phytochemicals were reported to inhibit various CYPs, for instance, a natural product of *Curcuma longa* can inhibit CYP1A2, CYP3A4, CYP21A2, and CYP17A1 (Schwarz et al., 2011). Now an intriguing question arises: can cinnamon and clove EOs also restrict identical protein CYP51 and impede the production of ergosterol in pathogenic fungi?

Hence, in the present study, the dominant pathogens, which caused postharvest decay in bagging-free apples, were first isolated and identified. In addition, the inhibitory activities of cinnamon and clove EO as well as their application methods against the pathogens were examined both *in vitro* and *in vivo;* meanwhile, a combination of approaches including microscopic (SEM and TEM) and molecular (transcriptomic and docking analysis) investigations was further carried out to provide insights into their antifungal mechanism. The aim of this study was to provide theoretical reference on the potential use of EOs as a natural and efficient preservative for the control of postharvest diseases in the cultivation mode transformation of the apple industry.

## Materials and methods

### Materials and chemicals

Fresh apple fruits under the bagging-free cultivation pattern were harvested at commercial maturity and were transferred to the laboratory within 6 h. Uniform fruits free of defects and mechanical damage were selected and stored at 0 ± 0.5°C, 90% RH.

Pure-grade cinnamon EO (bark steam distillation; origin: China) and clove EO (bud distillation; origin: China) were purchased from Guangzhou Hengxin Spice Co., Ltd., Guangzhou, China, and stored in the dark, at room temperature. The cinnamon and clove EO were analyzed on an Agilent gas chromatography–mass spectrometry (GC-MS) 7890 column (30 m × 0.25 mm × 0.25 μm). Helium was used as carrier gas. The injection volume was 1 μL, and the injector temperature was set at 250°C. The oven temperature was programmed at 50°C for 2 min, raised to 260°C at a rate of 5°C/min, and maintained for 10 min. In the full-scan mode, electron ionization mass spectra were recorded at 70 eV electron energy with a mass range of 10–550 Da. The temperatures of the interface, ion source, and quadrupole were held at 280, 230, and 150°C, respectively. The main components of EOs were assigned by comparing their relative retention time and matching their mass spectra characteristic features with the mass spectral library (Wiley Register TM of Mass Spectral Data). The main composition of cinnamon and clove EO is given in Table 1.

**Table 1:** Chemical compositions of cinnamon and clove EO.

**No**.	**Components**	**RT^a^ (s)**	**Percentage (%)**
**Cinnamon EO**			
1	*Trans*-cinnamaldehyde	553.74	82.228
2	Benzaldehyde	274.62	7.907
3	2,4-decadienal	587.52	3.174
4	Phenethyl acetate	532.32	1.387
5	Camphorene	265.56	0.714
6	4-isopropyltoluene	328.56	0.706
7	2-carbitol	464.16	0.688
8	Salicylaldehyde	347.82	0.612
9	O-methoxybenzaldehyde	521.10	0.506
10	O-methoxycinnamaldehyde	755.64	0.387
11	*Trans*-2-decenal	539.34	0.385
12	Nonanal	398.28	0.350
13	Eugenol	617.10	0.301
14	α-ylangene	733.14	0.228
15	2-undecenal	624.30	0.221
16	Limonene	332.94	0.175
17	Eucalyptol	336.36	0.031
**Clove EO**			
1	Eugenol	615.66	78.952
2	Eugenol acetate	740.16	17.892
3	β-caryophyllene	674.76	2.059
4	α-caryophyllene	702.78	0.726
5	Oxetene	798.66	0.281
6	α-Humulene	756.21	0.068
7	Methyleugenol	718.92	0.022

RT^a^, retention time.

### Survey of postharvest diseases

Apples were stored at 25°C after picking, and disease incidence was measured at 20, 40, and 60 days. Each treatment included three replicates, and each replicate consisted of 40 apples.

### Isolation, purification, and identification of pathogens

Isolation of the pathogens was carried out based on our previous method (Wang et al., 2020). Diseased fruits were surface-sterilized with 75% ethanol for the 30 s and 1% sodium hypochlorite (NaOCl) for 1 min. The sterilized tissues were rinsed three times with sterilized water, placed on potato dextrose agar (PDA) medium containing 0.02% streptomycin sulfate, and incubated in the dark at 25°C. Isolated fungal colonies were subcultured by the hyphal tip transferring technique until the pure culture was obtained. The two mold strains were obtained and marked as DH1 and HF2. After incubation for 7–10 days, the morphology of colonies and conidia of the two strains was observed and recorded. The isolated pathogens were assayed its pathogenicity in healthy apples. Furthermore, the pathogens were isolated again from diseased fruit using the earlier tissue separation method. Finally, the pathogen was subcultivated on PDA and stored at 4°C for consequent identification.

The genomic DNA of mycelia was extracted using CTAB (cetyltrimethylammonium bromide) method (Wang et al., 2020). The internal transcribed spacer (ITS) sequence of the rDNA was amplified using the primer ITS1 (5′-TCCGTAGGTGAACCTGCGG-3′) and ITS4 (5′-TCCTCCGCTTATTGATATGC−3′), and thermal cycling was set a predenaturation step at 94°C for 5 min, denaturation at 94°C for 40 s, annealing at 58°C for 40 s, following by 35 cycles of extension at 72°C for 1 min, and a final elongation step at 72°C for 10 min.

Polymerase chain reaction (PCR) products were purified and sequenced by Shanghai Boshang Biological Technology Co., Ltd. (Shanghai, China). To assess similarity, multiple related sequences were aligned by BLAST in the NCBI database. A phylogenetic tree was constructed using the neighbor-joining method in MEGA 5.2 software. *Torreya grandis* (AF259277.1) and *Pestalotiopsis microspora* (KF941280.1) were used as the out-group for DH1 and HF2, respectively. Bootstrapping was performed with 1,000 replicates to evaluate the significant internal branches in the tree.

### Mycelial growth inhibition testing *in vitro*

#### Gas diffusion test

A mycelial plug with a size of 7 mm from 7 days of actively growing culture was priorly inoculated onto the center of the bottom of the Petri dishes with 12.5 mL of PDA. Subsequently, a sterilized filter paper was attached to the center of the inner side of the plate lid with different amounts of EOs (1.5, 2.25, 3, and 3.75 μL) added; then, the plate lid was quickly covered, and 37.5 mL air space was offered to obtain final concentrations of 40, 60, 80, and 100 μL L^−1^ of air (v/v). PDA plates without EO were used as controls. All plates were sealed with laboratory parafilm to prevent leakage of EO vapor, kept in an inverted position, and incubated at 26°C for 4 days. The radical growth diameters of each treatment were measured using a digital vernier caliper in triplicate.

#### Solid diffusion test

Following our previous procedures with some minor modifications (Wang et al., 2019), 9.6, 19.2, 28.8, and 38.4 μL of pure EO were dispersed using 8 mL Tween 80 (0.2% v/v) while mixing with a high-speed homogenizer (IKA-ULTRATURRAX T25 basic, IKA Works, Inc., Wilmington, NC, USA) at 14,000 rpm for 4 min. Then, 40 mL PDA was added (48 mL total) immediately before it was poured into the glass Petri dishes (15 mL/plate, in triplicate) to obtain a final concentration of 200, 400, 600, and 800 μL L^−1^. The control was prepared similarly with Tween 80 alone. Afterward, mycelia was inoculated in the center of each plate. All plates were sealed with laboratory parafilm to avoid EO evaporation, kept in an inverted position, and incubated at 26°C for 4 days. The radical growth diameters of each treatment were measured using a digital vernier caliper in triplicate.

The lowest concentration of the EOs at which there was no strain growth for 48 h was defined as the minimum inhibitory concentration (MIC).

### Antifungal assays *in vivo*

The fungal inoculation was performed according to the previous research (Zhou et al., 2018). Apple fruits were sterilized with 2% sodium hypochlorite for 2 min and air-dried at room temperature. Each fruit was wounded (3 mm deep and 3 mm wide) at its equator using a sterile nail. Then, 10 μL of the spore suspension at 1.0 × 10^8^ spores L^−1^ were evenly inoculated into the puncture wounds (“Fuji” and “Orin” apples were inoculated with *B. dothidea* and *C. gloeosporioides*, respectively). The volume ratio of the EO and the container (μL L^−1^) was used to represent the vapor concentration. Based on our preliminary experiments, 60 μL L^−1^ EO were placed on filter paper. During EO vapor treatments, apple fruits were placed in a container of 6.5 L volume sealed with PVC cling film to make it evaporate naturally and stored in a 95% relative humidity incubator at 25°C. Samples for lesion diameter assay were recorded daily. Each treatment contained three replicates, and the whole experiment was performed twice.

### SEM and TEM assays

The scanning electron microscopy (SEM) and transmission electron microscopy (TEM) assays were carried out based on some previous studies (Li et al., 2014; Zhang et al., 2016). In brief, the spore suspension of *B. dothidea* and *C. gloeosporioides* was obtained from 4-day-old cultures by adding 10 mL 0.9% NaCl solution to each Petri dish and gently scraping the mycelial surface three times with a sterile L-shaped spreader to free the spores. A 1 mL spore suspension (1 × 10^7^ CFU mL^−1^) was added to 150 mL PDB medium and incubated at 26°C shaking for 2 days. The fully emulsified cinnamon and clove EO by Tween 80 solution (0.2% v/v) were added to the shake flask of *B. dothidea* and *C. gloeosporioides* to achieve the concentrations of their MIC, respectively. No EO added was set as the control. After that, all of the samples were incubated at 26°C for 12 h and collected by centrifugation at 4,000 rpm for 10 min. Then mycelial cells were washed three times with phosphate buffer solution (PBS). Each treatment was performed in triplicate.

For SEM assay, the cells were fixed with 2.5% glutaraldehyde and 4% formaldehyde for 4 h and were dehydrated with ethanol at gradient concentrations (15 min at 30, 50, 70, 80, and 90% and 20 min twice at 100%). Subsequently, the cells were freeze-dried, spray-gold, and visualized in an SEM (Sigma 300, ZEISS).

For the TEM assay, the mycelial cells were treated by ultra-thin sectioning and negatively stained (1% phosphotungstic acid, 5 min) and then directly examined in a TEM (MORADA-G2, Olympus, Japan).

### RNA-sequencing and bioinformatics analysis

Total RNA was extracted using TRIzol reagent (Invitrogen), and RNA quality and purity were assessed by Nanodrop spectrophotometer. In brief, rRNA was removed and mRNAs were fragmented, then transcribed mRNA fragments into first-strand cDNA, and followed by second-strand cDNA synthesis. After that, sequencing libraries were constructed and sequenced at Biomarker Technologies (Beijing, China) with an Illumina platform. Raw reads were filtered into high-quality clean reads used for subsequent bioinformatics analysis.

The gene expression levels were normalized by FPKM (fragments per kilobase of transcript per million fragments mapped). Genes with a *p*-value <0.05 and |log_2_FC| ≥ 1 were considered to be differentially expressed genes (DEGs). GO and KEGG enrichment analysis was performed on the identified DEGs. Only GO terms and KEGG pathways with FDR < 0.05 were statistically considered to be significantly enriched.

### Docking analysis

The CYP51 protein sequences of *Botryosphaeria dothidea* (KAF4313409.1) and *Colletotrichum gloeosporioides* (KAF4925022.1) were downloaded from the NCBI. The models of these proteins were built by the online tools SWISS-MODEL and assessed by using the program “PROCHECK” on the website of SAVES (https://nihserver.mbi.ucla.edu/SAVES/) for docking studies (Waterhouse et al., 2018). The 3D structures of the ligands including *trans*-cinnamaldehyde (CAS14371-10-9) and eugenol (CAS97-53-0) were downloaded from Pubmed. Molecular docking was performed using Autodock 4.2 software (Morris et al., 2009), and PyMOL (Delano, 2002) and LIGPLOT (Laskowski and Swindells, 2011) software were used to visualize and analyze the modes with the lowest binding score from the docking results.

### Statistical analysis

Data were listed as mean value ± standard deviation (SD) of three independent repeated experiments, as the interaction between treatment and experimental variables was not significant. All statistical analyses were performed using SPSS 16.0 software (IBM, Inc., NY, USA). One-way analysis of variance (ANOVA) was used to compare the three mean values. Mean separations were analyzed using Tukey's test correction. Differences at *p* < 0.05 were considered statistically significant.

## Results

### Occurrence of postharvest diseases

**Figure 1:**
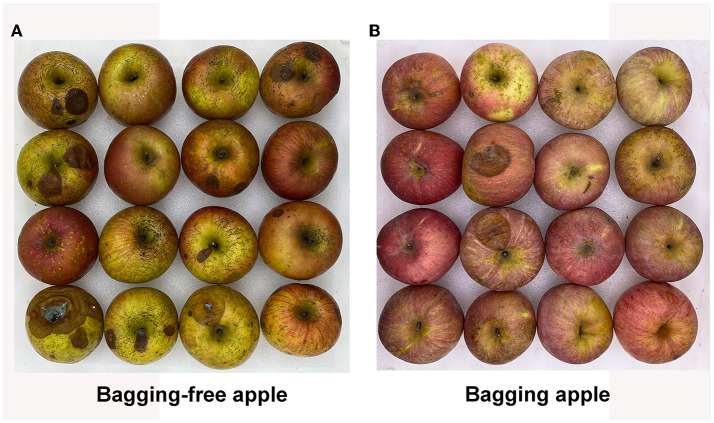
Different levels of fruit decay between bagging-free apple **(A)** and bagging apple **(B)** after 60 days of storage at 25°C.

We sampled apple fruits in Weihai, Yantai, and Taian, the main apple-producing areas in Shandong Province, and surveyed postharvest rot. As shown in Table 2 and Figure 1, regardless of the production area, the decay rate of apple fruits under the bagging-free cultivation mode was significantly higher than that under the bagging cultivation mode at the same time point (*p* < 0.05). Ring rot and anthracnose were the most important fungi-associated postharvest diseases of bagging-free apples (Table 3).

**Table 2:** Infection incidence of postharvest apple fruit.

**Treatments**	**Storage time (d)**	**Bagging-free cultivation mode**			**Bagging cultivation mode**		
		**Weihai**	**Yantai**	**Taian**	**Weihai**	**Yantai**	**Taian**
Decay rate (%)	20	2.8b	3.5b	7.5a	0c	0c	0c
	40	31.4b	34.3b	45.0a	3.0c	2.0c	2.5c
	60	40.0c	57.1b	66.0a	11.0d	8.0d	12.3d

Different lowercase letters indicate significant differences in the same row (*p* < 0.05).

**Table 3:** Occurrence of different diseases in bagging-free apple.

**Treatments**	**Disease incidence (%)**				
	**Anthracnose**	**Bitter pit**	**Black spot**	**Ring rot**	**Rust**
Weihai	1.11a	0c	0c	0.97a	0.4b
Yantai	0.88b	0c	0.05c	1.32a	0.72b
Taian	1.39b	0d	0d	1.53a	0.66c

Different lowercase letters indicate significant differences in the same row (*p* < 0.05).

### Isolation and identification of pathogens

**Figure 2:**
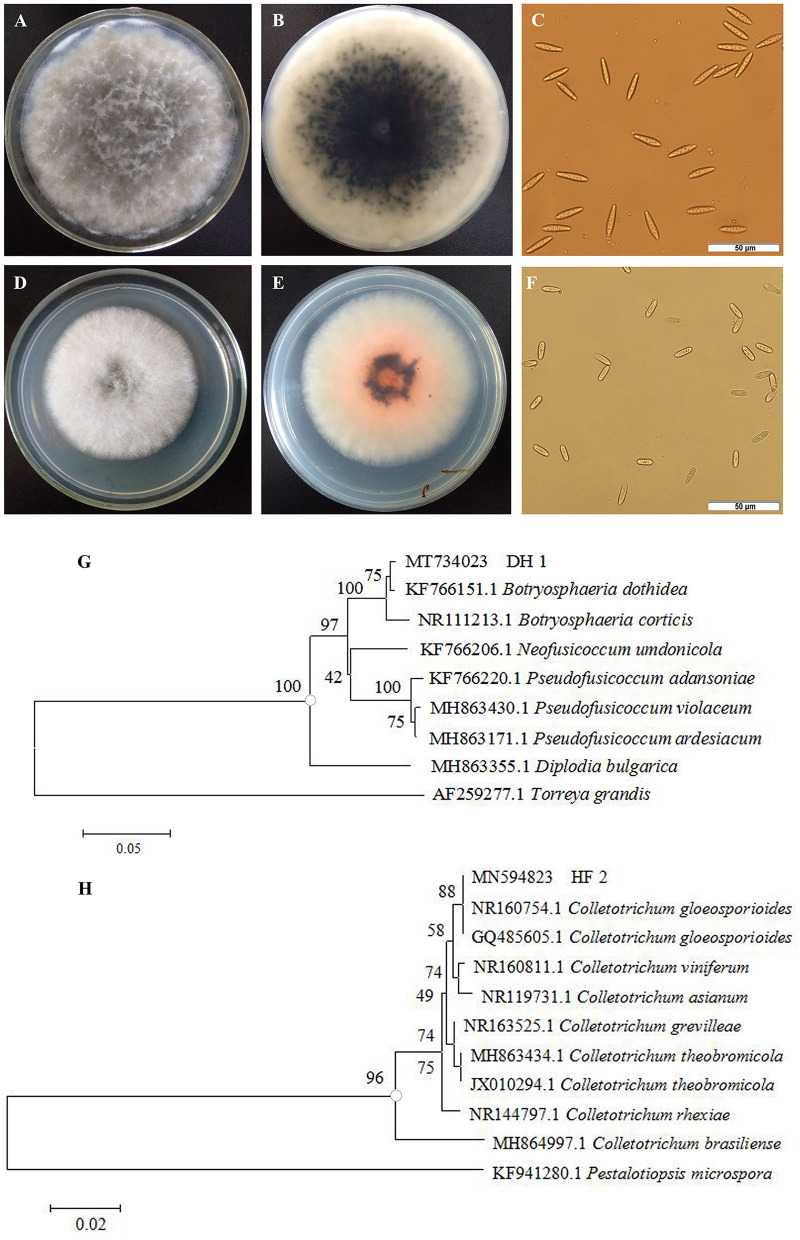
Morphological characteristics and phylogenetic tree of isolated pathogens. Colonies of pathogen DH1 **(A, B)** and HF2 **(D, E)**. Spores of pathogen DH1 **(C)** and HF2 **(F)**. Phylogenetic analysis of rDNA-ITS sequences obtained from the isolate DH1 **(G)** and HF2 **(H)** along with the reference sequences from NCBI.

Two strains of fungi were isolated from rotten non-bagging apples and were coded as DH1 and HF2. The colonial morphology and microscopic features of the isolated pathogens are shown in Figures 2A–F. The colony of the first pathogen DH1 was circular on PDA after 4-day incubation, and its color on the front of the plates was white to become dark gray. The aerial mycelia grew radically from the center to the surrounding area, and its texture was loose and cotton-like. Mycelia were hyaline and septate. The germinal spores were the single, colorless, rod-shaped, diameter of (15.0 – 29.5) × (5.1 – 7.6) μm (*n* = 40). The morphology of colonies and conidia was identical with *Botryosphaeria dothidea* (Wang et al., 2020). On the PDA medium of the second pathogen HF2, the colony was circular with regular edges. Mycelia were initially grayish white, and their texture was soft and villous. After 4–7 days, they turned dark gray with orange conidial masses. Under the microscope, we determined that conidia were hyaline and cylindrical to oblong, with 13.0–22.2 μm of length and 5.0–7.2 μm of width (*n* = 40). Morphological characteristics were in accordance with *Colletotrichum gloeosporioides* (Riera et al., 2019).

To further identify the two isolated pathogens, internal transcribed spacer (ITS) regions were sequenced. DNA sequences of these genes were deposited in GenBank with Accession Nos. MT734023 (DH1) and MN594823 (HF2), respectively. The homology sequences were analyzed with MEGA 5.02 software to construct a phylogenetic tree by the neighbor-joining method. Bootstrap values from the neighbor-joining method were determined. *Torreya grandis* (AF259277.1) and *Pestalotiopsis microspora* (KF941280.1) were used as the out-group for DH1 and HF2, respectively. In the phylogenetic tree, DH1 and other reference strain *B. dothidea* (KF766151.1) formed a clade with 100% bootstrap support (Figure 2G), while HF2 and two other reference strain *C. gloeosporioides* (NR160754.1, GQ485605.1) formed a clade with 100% bootstrap support (Figure 2H). Based on morphological features and molecular analysis, DH1 and HF2 were identified as *Botryosphaeria dothidea* (*B. dothidea*) and *Colletotrichum gloeosporioides* (*C. gloeosporioides*), respectively.

### Antifungal activity of EOs against *B. dothidea* and *C. gloeosporioides*

**Figure 3:**
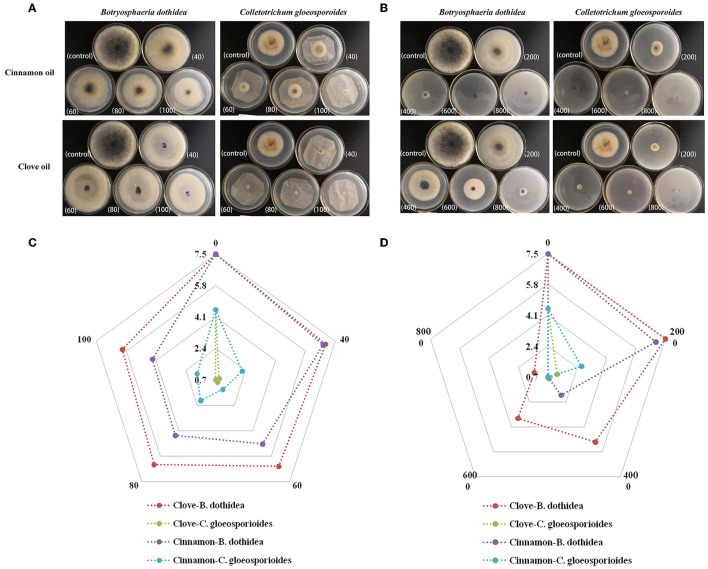
Mycelial growth inhibition of cinnamon and clove EOs against *B. dothidea* and *C. gloeosporioides*. **(A)** Morphological images of medium in EO vapor phase. **(B)** Morphological images of medium in EO contact phase. **(C)** Radar map of the radical growth diameters in EO vapor phase. **(D)** Radar map of the radical growth diameters in EO contact phase.

We further evaluated the inhibition of mycelial radical growth *in vitro* by fumigation and contact treatments. As presented in Figure 3, the inhibition zone diameter in all treatment exposure to EO was smaller than that in control at 25°C for 96 h. Lower concentrations were needed to inhibit the development of colony diameter by gaseous contact than by solution contact (*p* < 0.05). Both of the two EOs had stronger inhibitory effectiveness on *C. gloeosporioides* than that of *B. dothidea* by both solid and gas diffusion tests (*p* < 0.05), which indicated that *C. gloeosporioides* was more sensitive, while *B. dothidea* was more resistant to EO. In addition, the two EOs showed variable degrees of antifungal activity against the tested pathogens, which was verified by both fumigation and direct contact treatments. For *B. dothidea*, cinnamon EO exhibited an obviously stronger inhibition effect compared with clove EO; however, for *C. gloeosporioides*, cinnamon EO had poorer fungitoxic ability than clove EO. The MIC value of cinnamon and clove EO on *B. dothidea* and *C. gloeosporioides* by contact and vapor diffusion tests as presented in Table 4 confirmed the earlier statement.

**Table 4:** MIC values of cinnamon and clove EO on *B. dothidea* and *C. gloeosporioides*.

**Treatment**	**MIC (μL ·L^−1^)**
**Vapor phase**	
Cinnamon EO-*B. dothidea*	120
Cinnamon EO-*C. gloeosporioides*	80
Clove EO-*B. dothidea*	150
Clove EO-*C. gloeosporioides*	40
**Contact phase**	
Cinnamon EO-*B. dothidea*	400
Cinnamon EO-*C. gloeosporioides*	300
Clove EO-*B. dothidea*	600
Clove EO-*C. gloeosporioides*	200

### Effects of EOs on rot development in inoculated bagging-free apple

**Figure 4:**
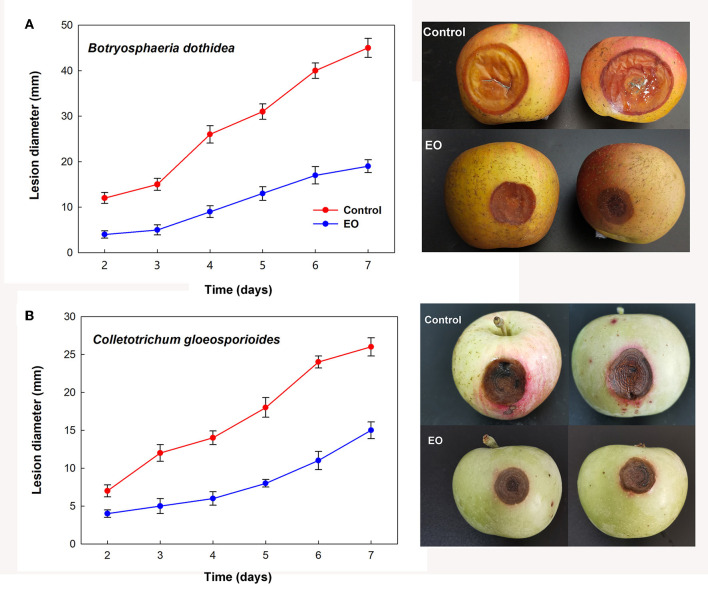
Controlling effect of cinnamon and clove EO on mold rot caused by *B. dothidea* **(A)** and *C. gloeosporioides* **(B)** in bagging-free apple. Bar represents the standard deviation of the means of three independent experiments. Lesions in apple fruit are also shown after 7 days of storage at 25°C.

Further investigation of the inhibition effects of EOs on the lesion diameter of fruit rot caused by *B. dothidea* and *C. gloeosporioides* demonstrated alleviated severity of corruption after treatment with cinnamon and clove EO fumigation, respectively. As shown in Figure 4, EO groups indicated significant decreases in bagging-free apple rot as compared to the control during the same period of storage after inoculation (*P* < 0.05).

### Effects of EOs on hyphal morphology and cellular ultrastructure

**Figure 5:**
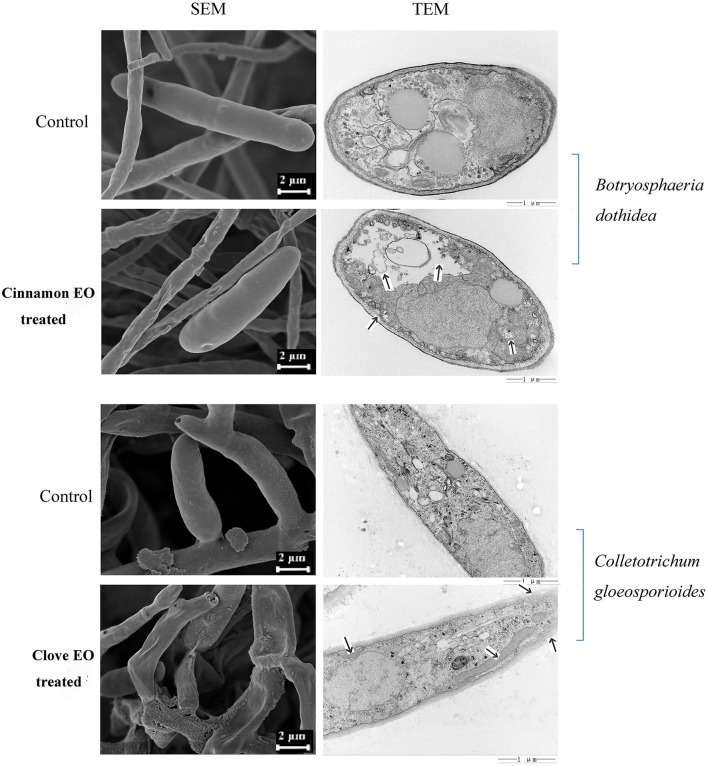
SEM and TEM micrograph of pathogen cells treated and untreated with EOs.

From the SEM images in Figure 5, we could intuitively observe the hyphal morphological alterations of *B. dothidea* and *C. gloeosporioides*. In the control groups, the mycelia were smooth, flat, and uniform, presenting a flourishing growth period. After 48 h EO treatment, the hyphae became folded, winding, rough, and even collapsed, indicating the destruction of the mycelium wall.

The effects of EOs on the cellular ultrastructure were observed by TEM. The control samples revealed uniform shapes in which all organelles had a normal and regular appearance and were clearly identified, while in the treated fungi, most organelles were indistinct and unidentifiable with deformed and disorganized mitochondria. Meanwhile, the cell wall was gradually becoming rough and villous, and the cell membrane was partially detached from the cell wall (Figure 5). SEM and TEM analyses partly manifested the mechanism of antimicrobial activity, but further investigation is required to reveal more gene-based changes during treatment with EO.

### Transcriptome analysis of *C. gloeosporioides* exposed to clove EO

#### The overall profile of gene transcription

Based on the results of antifungal activity analysis, we selected clove EO against *C. gloeosporioides* cells to further explore the potential antifungal mechanisms underlying the molecular response. High-throughput sequencing of the transcriptome was performed to screen the global gene expression profile changes of *C. gloeosporioides* with or without clove EO fumigation treatment (for 6 h at 1 × MIC concentration).

**Figure 6:**
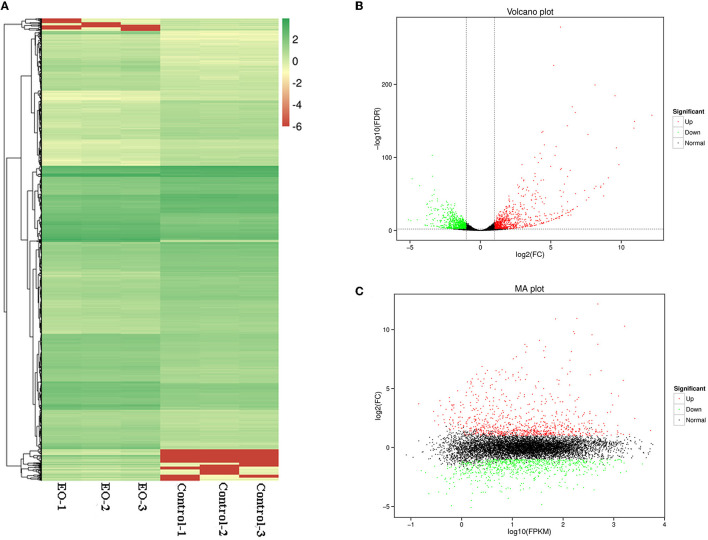
Differentially expressed genes (DEGs) analyzed in *C. gloeosporioides* cells for global comparison of transcription profiles (clove EO treatment vs. control). **(A)** Heatmap. **(B)** Volcano plot. **(C)** MA plot.

A total of 1,662 genes were identified as significantly differentially expressed genes (DEGs) (*p* < 0.05, |Log_2_FC| ≥ 1), with 857 genes upregulated and 805 genes downregulated. As shown in Figure 5, all the DEGs were displayed in Heatmap (Figure 6A), volcano plot (Figure 6B), and MA plot (Figure 6C) to be illustrated in a macroscopic view.

**Figure 7:**
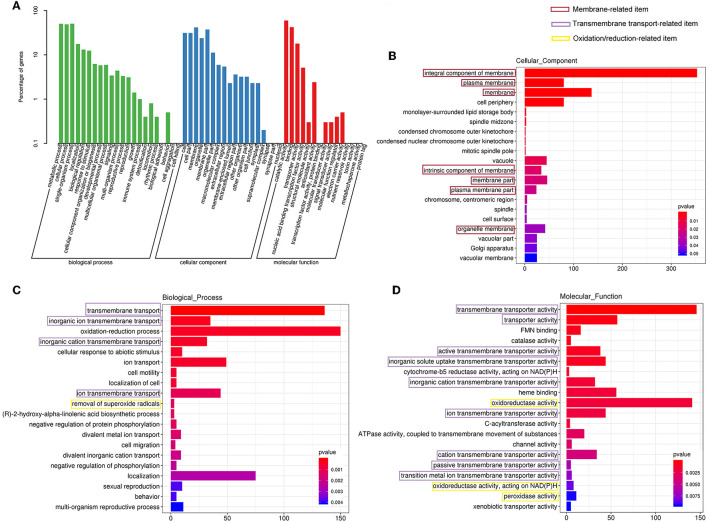
Gene Ontology (GO) analysis of the differentially expressed genes. **(A)** The GO classification. GO enrichment for cellular component **(B)**, biological process **(C)**, and molecular function **(D)** category, respectively.

Furthermore, all the DEGs were subjected to Gene Ontology (GO) annotation and KEGG enrichment analysis. As shown in Figure 7A, the DEGs were assigned to 19 biological processes, 15 cellular components, and 10 molecular functions. The top three categories of the biological process were the metabolic process (GO0008152), the ingle-organism process (GO0044699), and the cellular process (GO0009987). In the cellular component classification, the most represented categories were membrane (GO0016020), cell (GO0005623), and organelle (GO0043226). These results demonstrated that many DEGs were involved in the changes in cell membrane components, which was consistent with our observation of SEM and TEM. Catalytic activity (GO0003824), binding (GO0005488), and transporter activity (GO0005215) occupied the most variable categories of the molecular function classification.

**Figure 8:**
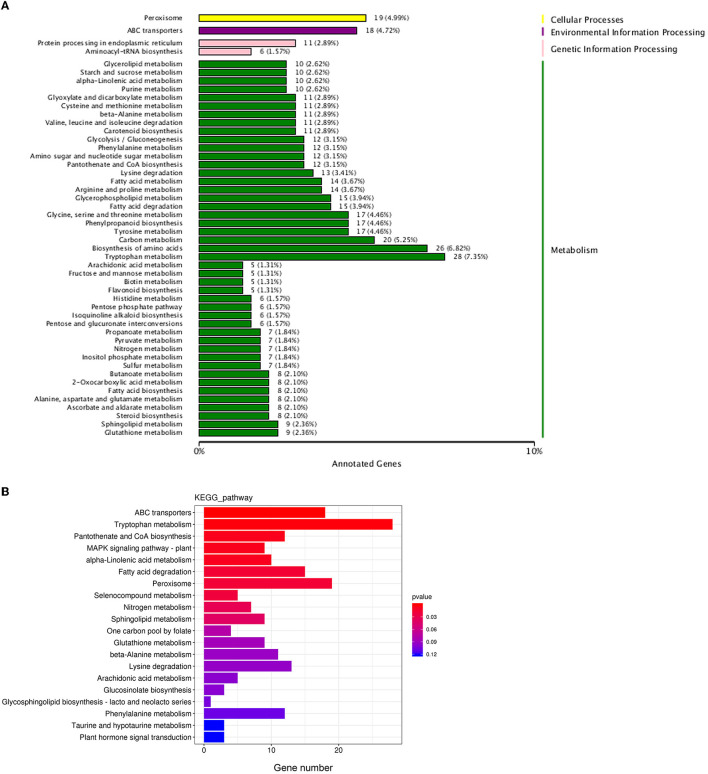
Statistical analysis of the differentially expressed genes in KEGG pathways. **(A)** The KEGG pathways annotation; **(B)** the enrichment of the differentially expressed genes in KEGG pathways.

Biological functions are vital to recognizing the antimicrobial mechanisms of EOs. Then, KEGG enrichment was performed to further explore the DEGs associated with the biological response pathways. In total, the DEGs were assigned to 49 KEGG annotation pathways, and the highly ranked terms were tryptophan metabolism (ko00380), peroxisome (ko04146), ABC transporters (ko02010), fatty acid degradation (ko00071), pantothenate and CoA biosynthesis (ko00770), lysine degradation (ko00310), and phenylalanine metabolism (ko00360), respectively (Figure 8A). From these data, most of the enrichment pathways were relevant to metabolism. Based on both GO annotation and KEGG enrichment, some crucial genes or pathways were screened out for further investigation as below.

#### Regulation of genes related to the cell membrane and cytoplasmic components

The GO enrichment analyses of the cellular component category manifested that membrane-associated items were mainly enriched (Figure 7B), and more genes were downregulated in these GO terms (Supplementary Figure 1D). Furthermore, other terms related to critical cytoplasmic components such as vacuole, Golgi apparatus, and spindle were also enriched (Figure 7B).

#### Regulation of genes related to cell transmembrane transport

A total of 18 items were involved in transport under the biological process and molecular function category in the GO enrichment analysis (Figures 7C, D). It could be seen that items related to transmembrane transport accounted for the greatest proportion in molecular function classification (Figure 7D), and most of the genes were downregulated (Supplementary Figure 1F), which was consistent with the response of cell membrane mentioned in the previous paragraph.

Meanwhile, as shown in the KEGG database, ATP-binding cassette (ABC) transport (ko02010), the most enriched pathway assigned to the environment information processing category (Figure 8B), was selected for further analysis. There were 18 DEGs in the ABC transport pathway with both upregulated and downregulated (Figure 8A). For example, POU3F3 (encoding POU domain, class 3, transcription factor 3, log_2_ FC = 9.84), *CH25H* (encoding cholesterol 25-hydroxylase, log_2_ FC = 1.76), and *TMEM258* (encoding transmembrane protein 258, log_2_ FC = 3.35) of ABCG2 subfamily (involved in the export of toxic compounds, organic anionic, and the translocation of various lipid molecules) were all upregulated. *Prdm1* (encoding PR domain zinc finger protein 1, log_2_ FC = −1.38), *CSH2* (encoding somatotropin family of hormones, log_2_ FC = −1.25), *AGA* (encoding aspartylglucosaminidase, log_2_ FC = −4.22) of ABCB1 subfamily (involved in the export of mitochondrial peptides, pheromone, and xenobiotics), and *ASIC2* (encoding decentering/epithelial sodium channel, log_2_ FC = −3.15), *RLF* (encoding Zn-15 related zinc finger protein, log_2_ FC = −2.74), and *ZNF137P* (encoding zinc finger protein 137, log_2_ FC = −4.73) of ABCG2 were all downregulated.

#### Regulation of genes related to the oxidation/reduction reaction

Gene Ontology enrichment analysis illustrated that the oxidation/reduction reaction of *C. gloeosporioides* cells was remarkably affected by clove EO treatment. As demonstrated in Figures 7C, D, oxidation–reduction process was enriched in the biological process and molecular function category. The expressions of about two-thirds of the DEGs involved in the oxidation/reduction reaction process were upregulated (Supplementary Figures S1B, C).

### Docking analysis of EOs constituents with CYP51 of pathogenic fungi

According to the TEM observation and the transcriptome analysis, it could be deduced that the presence of the EOs led to the alterations of the cell membrane composition and transmembrane transport function in the pathogenic fungi. To characterize the EO's antifungal mechanism more specifically and completely, molecular docking was used to explore the potential binding site of the inhibitory interaction. From the results of GC-MS analysis shown in Table 1, the main chemical components of cinnamon and clove EO were *trans*-cinnamaldehyde and eugenol, which have been reported as effective antimicrobial agents for foodborne pathogenic microorganisms in many studies. Here, *in silico* analysis studies could provide insight into the potential binding affinity of *trans*-cinnamaldehyde and eugenol with the key transmembrane protein CYP51.

#### Homology modeling

**Figure 9:**
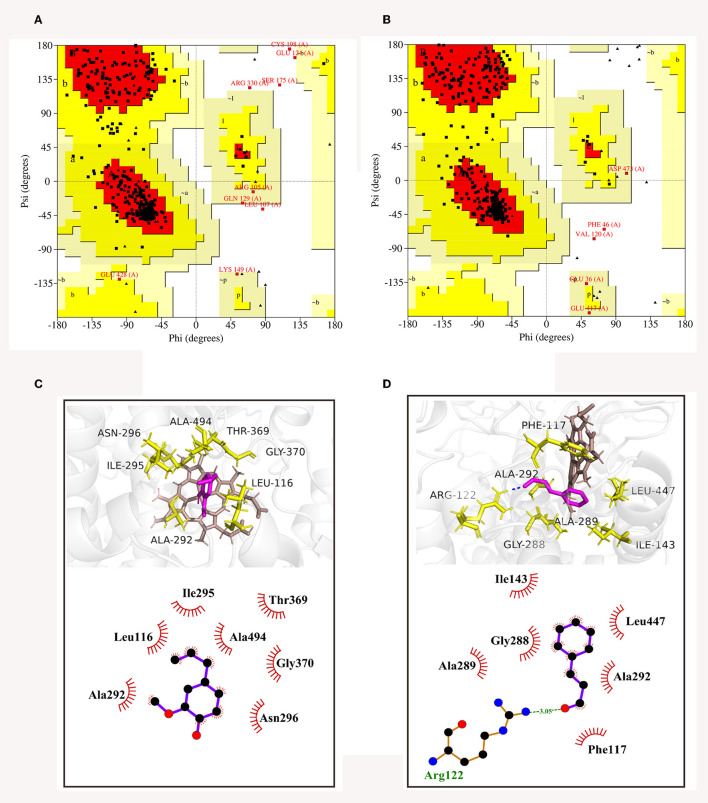
Ramachandran plots of CYP51 of *C. gloeosporioides* **(A)** and *B. dothidea* **(B)**, as well as the 3D and 2D diagrams of docked modes of CYP51 with *trans*-cinnamaldehyde **(C)** and eugenol **(D)**.

The 3D structures of CYP51 of *C. gloeosporioides* and *B. dothidea* have not yet been analyzed; therefore, based on their amino acid sequences, homology modeling was utilized to obtain the best models of the two CYP51 proteins. Then, model qualities were assessed by the online tool SAVES. As shown in Ramachandran plots, CYP51 of *C. gloeosporioides* had 88.2% of the amino acid residues in the most favored regions (red areas) and 9.6% in the additional allowed regions (yellow areas) (Figure 9A); CYP51 of *B. dothidea* had 88.9% of the amino acid residues in the most favored regions (red areas), and 9.9% are in additional allowed regions (yellow areas) (Figure 9B); the proportion of total amino acids in the reasonable range is 97.8 and 98.8%, respectively (Figures 9A, B), which were indications of high quality for a model.

#### *In silico* analysis

The optimal binding conformations of eugenol with CYP51 of *C. gloeosporioides* and *trans*-cinnamaldehyde with CYP51 of *B. dothidea* were shown in Figures 9C, D, with a binding score of −4.45 and −4.69 kJ mol^−1^, respectively. Eugenol was well-embedded into a cavity in the vicinity of the active site, showing a hydrophobic behavior with the key amino acid residues which included Leu116, Ala292, Ile295, Asn296, Thr369, Gly370, and Ala494 (Figure 9C). *Trans*-cinnamaldehyde formed one hydrogen bond with Arg122 and interacted with other residues (Phe117, Ile143, Gly288, Ala289, Ala292, and Leu447) *via* the hydrophobic effect (Figure 9D). It was suggested that *trans*-cinnamaldehyde showed a better *in silico* affinity toward CYP51 than eugenol because of a higher binding score and additional hydrogen bond.

## Discussion

Because of the relatively low rot rate of bagging apples under low temperatures and controlled atmosphere conditions, a previous study has been largely focused on postharvest physiological diseases, for instance, superficial scald and bitter pit (Susan and Watkins, 2012; Jarolmasjed et al., 2016). In this study, a higher rot degree was found in apple fruit under bagging-free cultivation mode (Figure 1; Table 2); thus, pathological research related to the control of fungal decay during the conservation period is vital to raise the commercial value of bagging-free fresh fruit.

The advantage of EOs is their bioactivity in the vapor phase, making postharvest sterilization processing more convenient for the stored commodity (Tzortzakis, 2009). It was found that EOs of the volatile phase were effective at a very low concentration and were beneficial in limiting the spread of the pathogen by lowering the spore load (Chutia et al., 2009). The results of the present study indicated that cinnamon and clove EO in vapor and contact phases both showed antifungal activity on the mycelial growth of *B. dothidea* and *C. gloeosporioides* in a concentration-dependent manner *in vitro* condition, and the volatile phase exhibited more toxic than the contact (Figure 3). SEM also demonstrated large and abnormal alterations in hyphal morphology, indicating the degeneration of fungal hyphae (Figure 5). Furthermore, *B. dothidea* and *C. gloeosporioides* had variable resistance against cinnamon and clove EO, which could be related with distinct sensitivity of pathogenic cells to the different types and amounts of EO components. Cinnamon and clove EO are classified as “generally regarded as safe” (GRAS) by the United State Food and Drug Administration (Hammer et al., 2006). For the *in vivo* test, the EOs vapor treatment also alleviated the severity of fruit rot in artificially infected bagging-free apples (Figure 4). In the future, the synergistic or additive effects between the two EOs could be further investigated to reduce the active doses needed to control postharvest rot for bagging-free apple.

The fungal cell membrane consists of a semi-permeable lipid bilayer that protects the integrity of the cell along with maintaining the cell shape and regulates the transport of materials entering and exiting the cell. It is the main target for the fungistatic action of EOs due to their lipophilicity (Burt, 2004; Paul et al., 2011). For example, tea tree EO destroyed membrane integrity and increases the permeability of *Botrytis cinerea*, resulting in ion leakage and membrane dysfunction (Yu et al., 2015). Bayer et al. (2000) and Pasqua et al. (2007) demonstrated a strong decrease in the unsaturated fatty acids and a high degree of saturated fatty acids in the fungal membrane in essential oil-treated bacteria, causing a decrease in membrane fluidity and a consequent increase in its rigidity. In our previous study, clove EO exposure to *C. gloeosporioides* caused leakage of intracellular proteins and nucleic acids and ultimately cell lysis (Wang et al., 2019). Our TEM observation found the shriveled, ruptured, or disappeared plasmalemma, the loss or disappearance of cytoplasm, and the extrusion of abundant material from the outside of the cell wall (Figure 5). Transcriptome had been widely used to conduct a preliminary analysis of the gene-based mechanisms by which active substances inhibit pathogens, such as thymol against *Fusarium oxysporum* (Liu et al., 2022) and nerol against *Ceratocystis fimbriata* (Li X. Z. et al., 2021). The GO terms assigned by DEGs related to membrane occupied the most represented categories of the cellular component classification (Figure 7B), and the majority of genes were downregulated (Supplementary Figure S1D), inferring that these genes might be the target of clove EO to destroy the cellular membrane. The membrane undertakes the function of transmembrane transport of various substances; after treatment of clove EO, the genes coding the structure and component of the cell membrane were downregulated, which may decrease the overall capacity of cellular molecules transport. ABC proteins, which were exclusively found in both prokaryotes and eukaryotes, form a large subfamily of ATP-dependent transporters that participate in mediating cellular import and export processes and play vital physiological roles (Wilkens, 2015). Approximately 18 DEGs associated with ABC transporters were detected (Figure 8A), implying that the regulation of materials transport (such as lipids, toxins, minerals, and organic ions) altered significantly after clove EO treatment. Ergosterol is the major sterol component of the fungal cell membrane, helping to maintain cell function and integrity, and is considered the fundamental target of antifungal drugs (Pinto et al., 2013). As mentioned earlier, dill EO could cause a considerable reduction in ergosterol quantity (Tian et al., 2012). CYP51 belongs to a main transmembrane protein and serves as the key enzyme in the fungi ergosterol biosynthesis pathway (Zhang et al., 2019). The suitability of CYP51 as an antifungal azole target has been discussed in many previous studies (Song et al., 2018); however, there is still limited information about the relationship between the antifungal action of EOs and CYP51. To investigate the inhibitory mechanism of cinnamon and clove EO on *B. dothidea* and *C. gloeosporioides*, relevant protein-molecular interactions were first evaluated by *in silico* analysis. According to the molecular docking results, we noticed high binding scores of *trans*-cinnamaldehyde and eugenol with the protein CYP51 of the two identified pathogenic fungi. *Trans*-cinnamaldehyde and eugenol entered the active pocket of the protein and led to instability of the catalytic region, which further attenuated CYP51 activity and decreased ergosterol content. In CYP51 of *C. gloeosporioides*, eugenol was well-embedded into a cavity in the vicinity of the active site, the key residues of which included Leu116, Ala292, Ile295, Asn296, Thr369, Gly370, and Ala494 (Figure 9C). In CYP51 of *B. dothidea, trans*-cinnamaldehyde formed one hydrogen bond with Arg122 and showed a hydrophobic behavior with other residues (Phe117, Ile143, Gly288, Ala289, Ala292, and Leu447) (Figure 9D). The hydrogen bonding is considered to play a major role in the protein-molecular interactions (Gerdt et al., 2015). In this study, *trans*-cinnamaldehyde showed a better *in silico* affinity toward CYP51 than eugenol because of a higher binding score and additional hydrogen bond. Our data confirmed that the inhibitory mechanism of EOs on the cell membrane of pathogens might involve the interaction of antifungal components with the CYP51 protein and subsequent interference of ergosterol biosynthesis. These findings strongly supported EOs inhibited the pathogen microorganism through loss of cytoplasmic membrane integrity and function.

One of the earliest and most prominent fungi defense responses is an oxidative burst. Our transcriptome data also showed that the oxidation/reduction reaction of *C. gloeosporioides* cells was remarkably affected by clove EO treatment (Figures 7C, D), and the expression of the majority of the DEGs involved was upregulated (Supplementary Figures S1B, C), which corresponds to the findings of Guo et al. (2019) and Wu et al. (2022). This indicated that clove EO caused some degree of oxidative stress and activated defensive function against stress response to enhance the antioxidant capacity of *C. gloeosporioides*, but some other antifungal agents, such as iturin A and acriflavine, downregulated most genes involved in oxidation–reduction reaction of fungi (Persinoti et al., 2014; Jiang et al., 2020). This discrepancy suggested that the oxidation/reduction process might respond distinctly under various stimuli or stress.

In addition, the clove EO showed a marked disruption of the cell wall and major organelles such as mitochondria, Golgi apparatus, vacuole, and disorder of biological functions including metabolism of crucial materials (Figures 7, 8). Overall, EOs contribute to antimicrobial activity through diverse modes of action, which need further investigation.

Despite the prominent preservative potency of EOs in the food system, some limitations have been recognized in their practical application, such as negative effect on organoleptic properties, volatility, low water solubility, and low stability, which prevent their large-scale practical utilization (Akash et al., 2021). Hence, specific delivery systems are required for a gradual release of EO aroma compatible with food-based applications. Nanoencapsulation (Das et al., 2021), active packaging (Li et al., 2018), and polymer-based coating (Guerra et al., 2016) are promising delivery strategies of EOs that help ease dispersion with consistent antimicrobial action and enhancement in food shelf life. Furthermore, controlled release behavior minimizes EO's impact on food organoleptic attributes and showed better diffusion kinetics.

The increased use of EOs has also raised serious concerns with respect to their eventual adverse health and environmental effects, though this is still waiting to be confirmed (Woolf, 1999). In the national standards for food safety of China, EO is the designation for ingredients approved as food additives. In America, EO is considered “Generally Recognized as Safe” (GRAS) by the Food and Drug Administration (FDA), but the documents do not include dosages that are considered safe. In fact, there was evidence that when EOs are inappropriately used, they can give adverse effects in humans, such as skin irritation, headache, and nausea (Fandohan et al., 2008). Burdock and Carabin (2009) reported a non-observed adverse effect level (NOAEL) for *Coriandrum sativum* L. essential oil of ~160 mg/kg/day in rats, being considered safe at the present concentration used in food. Consumption of horticultural commodities treated with these oils is likely to be safe for humans if the recommended oil concentration is respected. Current information indicates that EOs are safe for the consumer and the environment with a few qualifications (Isman, 2000; Antunes and Cavaco, 2010). Considering the food-based industrial relevance of EOs, possible toxicological studies in a mammalian system and the market value of EOs need to be further investigated for their potential application as eco-friendly smart green preservatives in the food and agriculture industries.

## Conclusion

In the current study, the results demonstrated that the main pathogens causing postharvest decay of bagging-free apples were *B. dothidea* and *C. gloeosporioides*. *In vitro* and *in vivo* trials indicated that cinnamon and clove EOs fumigation effectively limited fungal growth and reduced rot. The two identified organisms had variable resistance against the tested Eos; consequently, cinnamon and clove EOs will be further studied for their synergistic effects on the control of postharvest spoilage in bagging-free apples. Morphological, transcriptomic, and docking analyses suggested that EOs reduced the component synthesis and activity of fungal cell membranes and disturbed the biological function of cytoplasmic components as a whole. In a word, cinnamon and clove EOs are potential biocontrol agent candidates for preventing and controlling diseases of bagging-free apples, but detailed examinations of the biological activity and undesirable effects on the postharvest fruit or on human health and the environment require further investigations.

## Data availability statement

The datasets presented in this study can be found in online repositories. The names of the repository/repositories and accession number(s) can be found in the article/Supplementary material.

## Author contributions

DW: methodology, writing—original draft, and visualization. GW: investigation and formal analysis. JW: resources, investigation, and writing—reviewing and editing. HZ: conceptualization, methodology, resources, and writing—reviewing and editing. XX: supervision, project administration, and funding acquisition. All authors contributed to the article and approved the submitted version.

## Supplementary material

Supplementary Figure S1 GO enrichment of up- **(A–C)** and down- **(D–F)** regulated genes for cellular component, biological process, and molecular function category.
