# Supplementary figures and images for: Inhibitory effect and underlying mechanism of cinnamon and clove essential oils on Botryosphaeria dothidea and Colletotrichum gloeosporioides causing rots in postharvest bagging-free apple fruits
Source: Front Microbiol. 2023 Feb 27;14:1109028. doi: 10.3389/fmicb.2023.1109028 (PMC10008952; doi:10.3389/fmicb.2023.1109028)

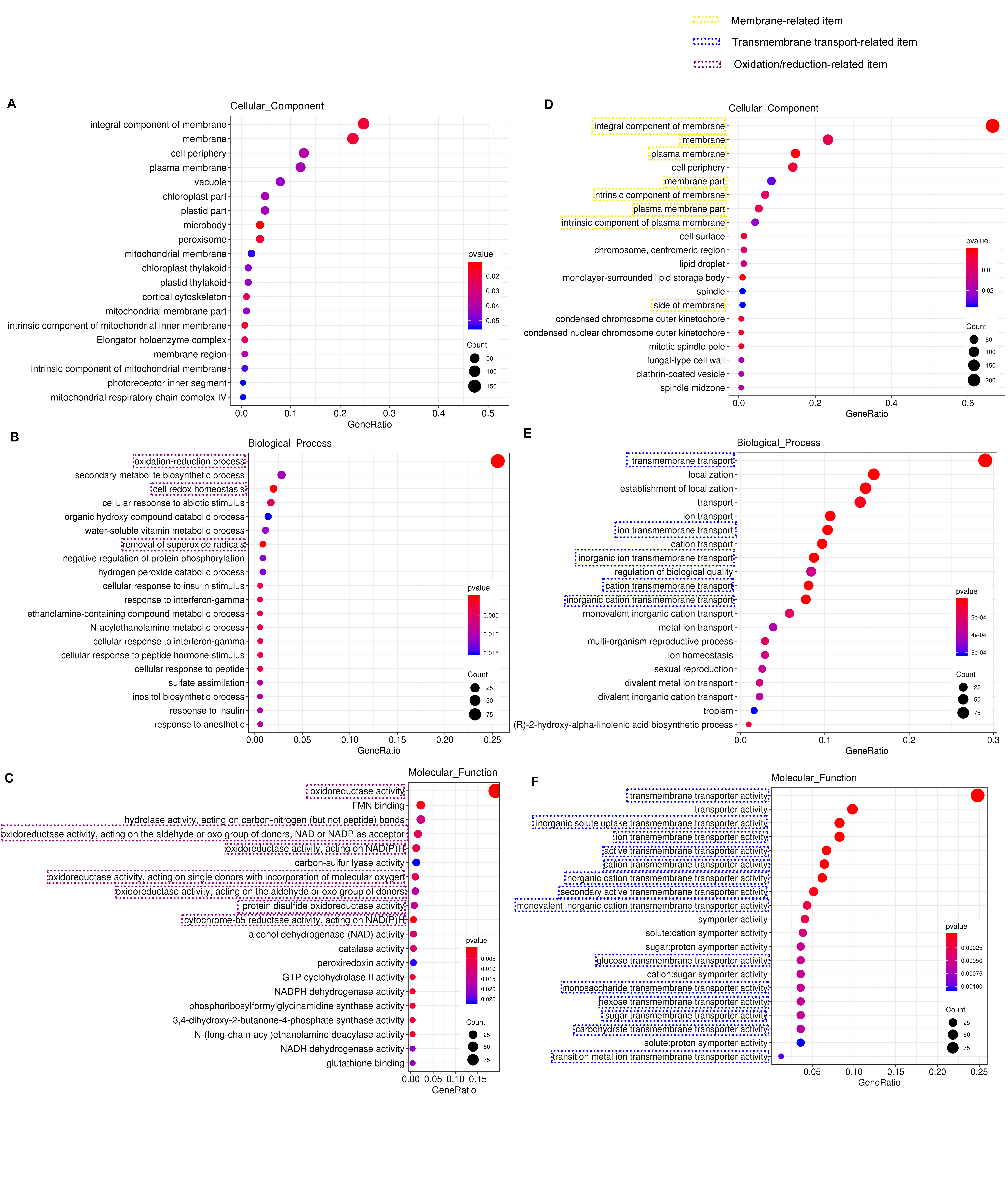

Supplement: Supplementary Figure S1 — GO enrichment of up- (A–C) and down- (D–F) regulated genes for cellular component, biological process, and molecular function category. [file Image_1.JPG]
